# Systematic review and meta-analysis of risk prediction models for anastomotic leak after gastric cancer surgery

**DOI:** 10.3389/fmed.2026.1855091

**Published:** 2026-06-12

**Authors:** Yang Sun, Rong Lu, Yunjuan Xu, Ping Jia, Zhenwei Liu

**Affiliations:** 1 North Sichuan Medical College, Nanchong, China; 2 Department of Neurosurgery, Sichuan Provincial People's Hospital, Affiliated Hospital of University of Electronic Science and Technology of China, Chengdu, Sichuan, China

**Keywords:** anastomotic leak, gastric cancer, meta-analysis, risk prediction model, systematic review

## Abstract

**Objective:**

The study aimed to evaluate risk prediction models for anastomotic leak (AL) after gastric cancer (GC) surgery through a systematic review and meta-analysis.

**Methods:**

We searched Chinese and English databases from inception to 25 December 2025. The Prediction model Risk Of Bias ASsessment Tool (PROBAST) was used for quality assessment. A meta-analysis was performed to pool the area under the receiver operating characteristic curve (AUC) values from externally validated studies. Subgroup analyses were performed to explore optimism bias. Predictors were qualitatively synthesized.

**Results:**

A total of 18 studies (15,783 patients) were included. The pooled external validation AUC was 0.795 (95% CI: 0.741–0.840; *I*^2^ = 54.6%). Internal validation showed a higher pooled AUC (0.856) than external validation (0.795), suggesting overfitting. Machine learning models had a higher pooled AUC (0.846) than regression models (0.775), but the difference was not significant (*p* = 0.094) and was based on only two studies. Models incorporating postoperative indicators performed significantly better than those using preoperative/intraoperative indicators (0.843 vs. 0.748, *p* = 0.0034), although this finding was also based on only two studies. We identified 61 predictors across eight categories; inflammatory markers (23%), nutritional markers (21.3%), and surgery-related factors (18%) were the most common. A total of 12 potential novel predictors were noted. Most studies reported calibration poorly, and only one study had a low risk of bias.

**Conclusion:**

Current AL prediction models show moderate-to-good discrimination but suffer from overfitting and methodological bias. Postoperative dynamic indicators may improve performance, but this requires confirmation. Future research should focus on prospective, multicenter, cross-regional external validation and better calibration reporting.

**Systematic review registration:**

https://www.crd.york.ac.uk/prospero/display_record.php?RecordID=272725, PROSPERO (CRD420251272725).

## 1 Background

Gastric cancer (GC) ranks as the fifth most common cancer worldwide, accounting for 4.9% of all new cancer cases (1). Radical gastrectomy is the treatment of choice for GC (2, 3); however, anastomotic leak (AL) remains one of the most devastating postoperative complications, with a reported incidence of 2.1–14.6% and a mortality rate of up to 50% (4). In recent years, risk prediction models for AL after gastric cancer surgery have proliferated rapidly, but their quality and applicability have not been systematically evaluated. To clarify the predictive performance of existing models, identify core predictors, and guide future investigation, we performed a systematic review and meta-analysis of studies developing and/or validating AL risk prediction models after gastrectomy.

## 2 Methods

The study protocol was registered with PROSPERO (registration number: CRD420251272725).

### 2.1 Inclusion and exclusion criteria

Studies were included if they met the following criteria: (1) patients aged ≥18 years who developed anastomotic leak (AL) after gastric cancer surgery, irrespective of tumor stage or surgical approach; (2) prospective, cohort, case–control, or retrospective studies; (3) studies that developed a prediction model and reported its performance metrics; (4) anastomotic leak after gastric cancer surgery as the outcome of interest; and (5) articles published in Chinese or English.

Studies were excluded if they: (1) did not develop a prediction model; (2) were duplicate publications; (3) were available only as abstracts; (4) lacked accessible full texts or sufficient data for extraction; (5) were reviews, case reports, conference abstracts, or similar non-original publications; or (6) did not report model performance metrics.

### 2.2 Search strategy

We systematically searched the following databases from inception through 25 December 2025: China National Knowledge Infrastructure (CNKI), the VIP database, Wanfang Data, China Biology Medicine disc (CBM), Web of Science, PubMed, CINAHL, Embase, and the Cochrane Library. The search targeted studies that developed risk prediction models for anastomotic leak after gastric cancer surgery. A combination of subject headings and free-text terms was employed. The search strategy used for PubMed is detailed in Table 1.

**Table 1:** Search strategy used in PubMed.

Item	Search strategy
#1	“Stomach Neoplasms”[Mesh] OR “Gastric Cancer”[tiab] OR “Stomach Cancer”[tiab] OR “Gastric Carcinoma”[tiab] OR “Gastrectomy”I[Mesh] OR gastrectomy[tiab]
#2	“AnastomoticLeak”[Mesh] OR “Anastomosis, Surgical/adverse effects”[Mesh] OR “Postoperative Complications”[Mesh] OR (anastomotic[tiab] OR anastomosis[tiab]) AND (leak*[tiab] OR fistula*[tiab] OR failure[tiab] OR complication*[tiab]) OR “AL”[tiab]
#3	“Risk Assessment”[Mesh] OR “Predictive Value of Tests”[Mesh] OR “Models, Statistical”[Mesh] OR “Nomograms”[Mesh] OR predict*[tiab] OR model[tiab] OR models[tiab] OR score[tiab] OR scores[tiab] OR rule[tiab] OR rules[tiab] OR calculator[tiab] OR nomogram*[tiab]
#4	#1 AND #2 AND #3

Detailed content of the other search strategies can be found in the Supplementary materials.

### 2.3 Literature screening and data extraction

A total of two authors (Y.S. and P.J.) independently screened the retrieved records. After removing duplicates, they reviewed titles and abstracts for initial eligibility. Full texts of potentially eligible studies were obtained and assessed, and reference lists were examined to identify additional relevant studies. Any disagreements were resolved by discussion or by consultation with a third researcher (Z.W.L.). A standardized data extraction form was developed based on the CHARMS checklist (5). The following information was extracted: study characteristics (first author, year of publication, country, study design, data source and study period, handling of missing data, total sample size, number of outcome events, outcome incidence, demographic characteristics, and study population) and model development details and performance metrics (model name, developer, number and content of predictors, timing of predictor collection, presentation format, AUC/C-index, calibration, and validation methods).

### 2.4 Literature quality assessment

The Prediction model Risk Of Bias ASsessment Tool (PROBAST) was used to evaluate the risk of bias and applicability of the included prediction models (6). For risk of bias, the tool comprises 20 signaling questions across four domains: Participants, predictors, outcome, and analysis. Each question is answered as “Yes,” “Probably Yes,” “No,” “Probably No,” or “No Information.” If at least one signaling question in a domain is answered “No” or “Probably No,” that domain is considered at high risk of bias. Applicability is assessed across three domains: Participants, predictors, and outcome. If any domain is rated as “high concern,” the model is judged to have high concern for applicability; if all domains are rated as “low concern,” the overall concern is considered low. A total of two authors (Y.S. and P.J.) independently performed the quality assessment. Disagreements were resolved through discussion or by adjudication with a third researcher (Z.W.L.).

### 2.5 Statistical methods

All analyses and plots were generated using R 4.5.2 and RStudio 2026.01.0 + 392. Key R packages used included gridExtra, ggrepel, meta, readxl, dplyr, ggplot2, forestplot, grid, and patchwork. For externally validated models, both fixed effects and random effects models were applied, with the random effects model serving as the primary method for pooling area under the receiver operating characteristic curve (AUC) values across studies. Given the bounded nature of the AUC, a logit transformation was employed to satisfy linear model assumptions: logit(AUC) = ln[AUC/(1 − AUC)] (7). The pooled logit(AUC) and its 95% confidence interval were obtained by pooling the logit-transformed AUC values and their standard errors (SEs). The expit function (inverse logit transformation) was then used to back-transform to the AUC scale: AUC_pooled = exp.[logit(AUC)_pooled]/{1 + exp.[logit(AUC)_pooled]}. Heterogeneity was assessed using the Q test, I^2^ statistic, and τ^2^ value, and funnel plots were generated to explore potential publication bias. Leave-one-out analysis was performed as a sensitivity analysis (8). Pre-specified subgroup analyses were conducted for three aspects. Comparisons of pooled AUCs between subgroups were performed using a random-effects meta-regression model, testing the interaction between the grouping variable and effect size, with a *p*-value of < 0.05 considered statistically significant. For internally validated models, scatter plots depicting sample size, events per variable (EPV), and AUC were constructed to examine common patterns and trends. For all models, predictors were systematically collated, categorized, quantitatively analyzed, and presented in bar charts.

## 3 Results

### 3.1 Study selection

**Figure 1:**
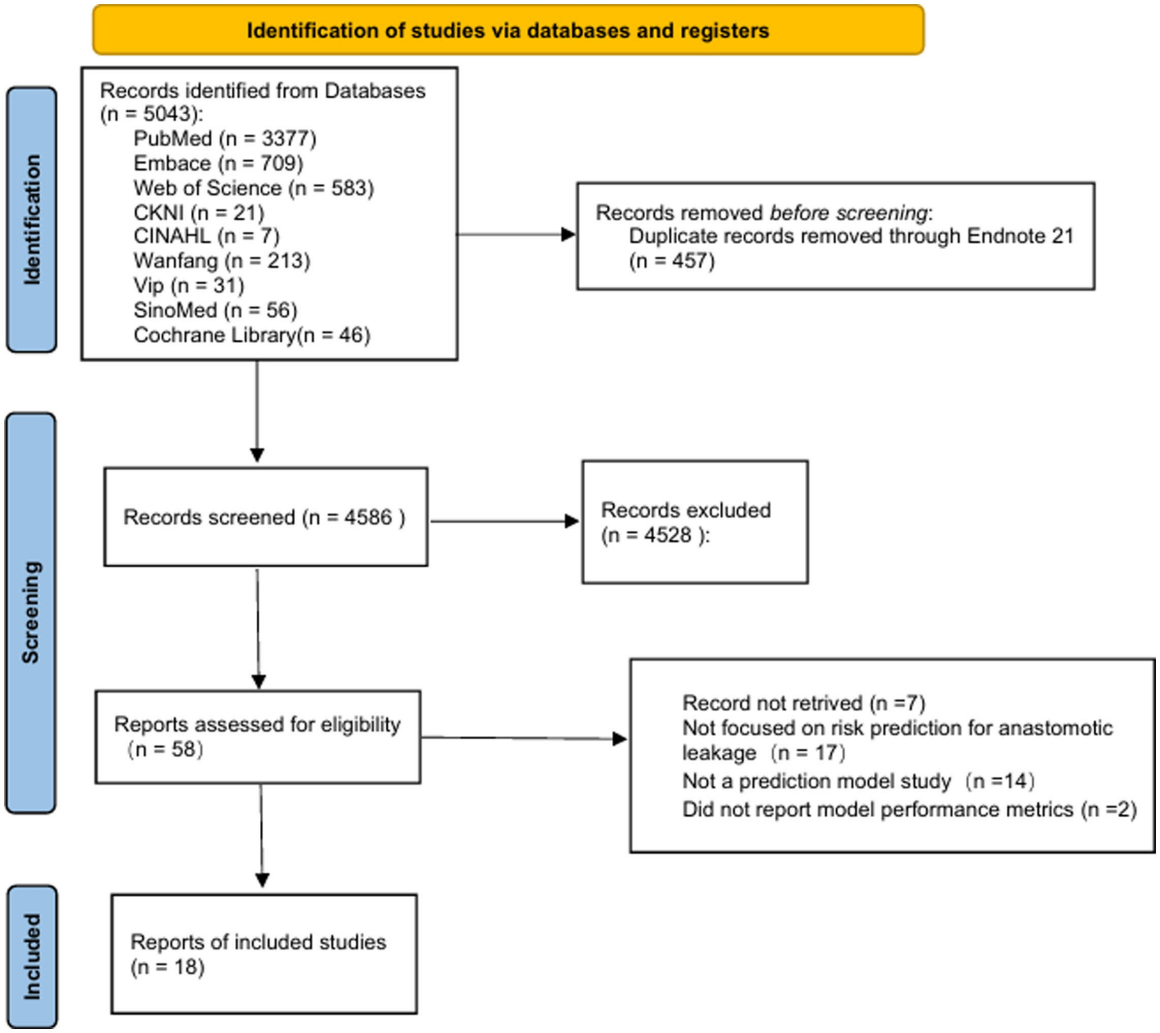
Flow diagram of study selection.

The initial search yielded 5,043 records. After removing 457 duplicates using EndNote 21, 4,586 records remained. A total of two reviewers (Y.S. and P.J.) screened the titles and abstracts and excluded studies that did not meet the inclusion criteria, as well as any additional duplicates not detected by the software. A total of 58 potentially eligible studies were retrieved for full-text review. Of these, seven studies were excluded because the full text could not be obtained, 17 were not prediction models for anastomotic leak, 14 did not address anastomotic leak as the outcome of interest, and two did not report model performance metrics. Ultimately, 18 studies (9–26) were included. The study selection process is presented in Figure 1.

### 3.2 Characteristics of the included studies

The basic characteristics of the 18 included studies are summarized in Table 2. Of these, 17 studies were conducted by authors in China and one in Italy, collectively involving 15,783 patients from 23 Chinese medical institutions and three Italian medical institutions. All prediction models targeted anastomotic leak (AL) after gastric cancer surgery, and the studies were published between 2017 and 2025. The study designs included eight retrospective cohort studies, six retrospective case–control studies, three prospective cohort studies, and one retrospective observational study.

**Table 2:** Basic information and characteristics of the included studies.

First Author/Year	Country	Study type	Missing data handling	Data source	AL cases/Sample size (%)	Study population
Zhang/2024 (13)	China	Retrospective cohort study	No missing	Hospital	35/639 5.40%	AL
Deng et al./2019 (12)	China	Retrospective case–control study	No missing	Hospital	75/800 9.38%	AL
Xia/2021 (11)	China	Retrospective case–control study	No missing	2 hospitals	34/815 4.18%	AL
Liu et al./2022 (10)	China	Retrospective cohort study	No missing	Hospital	Training set51/669 7.6% Validation set 223	AL
Man/2021 (9)	China	Retrospective case–control study	No missing	Hospital	17/136 12.5%	AL
Wang et al./2025 (14)	China	Retrospective cohort study	No missing	Hospital	13/238 5.40%	AL
Shao et al./2021 (15)	China	Retrospective observational study	YES	Hospital	36/1660 2.17%	AL
Shao et al./2025 (26)	China	Prospective cohort study	No missing	4 hospitals	13/512 2.54%	AL
Tang et al./2024 (16)	China	Retrospective case–control study	No missing	Hospital	31/275 11.27%	AL
Wang et al./2024 (17)	China	Retrospective cohort study	YES	Hospital	76/1508 5.0%	AL
Ma et al./2025 (18)	China	Retrospective cohort study	YES	2 hospitals	123/1587 7.8%	AL
Tu et al./2017 (19)	China	Retrospective cohort study	No missing	Hospital	50/3632 1.40%	AL
Shi et al./2022 (20)	China	Prospective cohort study	No missing	Hospital	24/326 7.4%	AL
Liu et al. /2022 (21)	China	Retrospective cohort study	No missing	Hospital	18/297 6.10%	AL
Cananz et al./2023 (22)	Italy	Prospective multicenter observational study	No missing	3 hospitals	14/297 4.60%	AL
Xu et al./2024 (23)	China	Retrospective case–control study	No missing	Hospital	21/476 4.40%	AL
Liao et al./2025 (24)	China	Retrospective cohort study	No missing	2 hospitals	88/1343 6.6%	AL
Zhao et al./2023 (25)	China	Retrospective study	No missing	Hospital	34/350 9.70%	AL

The prediction models employed a range of approaches, including logistic regression, nomograms, and random forest (RF) models. Among the studies, one performed external validation only, five conducted both internal and external validation, and 12 relied solely on internal validation. After deduplication, harmonization, and clinical classification, a total of 61 predictors were identified. Detailed model information and performance metrics are provided in Table 3.

**Table 3:** Model details and performance indicators.

Optimal model name	Developer	No. of predictors	Final predictors	Data collection time	Application form	Optimal model AUC/C-INDEX	Calibration	Validation method
Logistic regression model	Zhang Haofan et al.	6	(3), (17), (53), (30), (61), (2)	Preoperative and intraoperative	Nomogram	0.828	Qualitative: Good	Internal validation
Multivariate logistic regression model	Deng Fangyuan et al.	4	(1), (58), (18), (31)	Preoperative and intraoperative	Risk prediction equation score	0.734	Hosmer–Lemeshow test: *p* = 0.287	Internal validation
Nomogram prediction model	Xia Zhaoli et al.	4	(1), (19), (59), (32)	Preoperative and intraoperative	Nomogram	0.76	Qualitative: Good	Internal validation
Nomogram prediction model	Liu Ying et al.	5	(43), (1), (44), (20), (58)	Preoperative and intraoperative	Nomogram	(Internal validation): 0.760; (External validation): 0.741	Hosmer–Lemeshow test: *χ*^2^ = 6.079, *p* = 0.531	Both internal and external validation
Nomogram prediction model	Man Yifan et al.	3	(6), (4), (5)	Postoperative Day 5	Nomogram	0.988	Mean absolute error = 0.032	Internal Validation
Nomogram prediction model and Bayesian network model	Wang Yunfeng et al.	5	(33), (1), (21), (45), (3)	Preoperative and intraoperative	Nomogram	0.88	Decision curve analysis (DCA) showed net benefit	Internal validation
Random forest (RF) model	Shengli Shao et al.	10	(60), (1), (46), (47), (22), (41), (54), (55), (48), (34)	Preoperative and intraoperative	Web App	0.9	Not reported	Internal validation
Random forest (RF) model	Shengli Shao et al.	10	(60), (1), (46), (47), (22), (41), (54), (55), (48), (34)	Preoperative and intraoperative	Online web application	0.78	Qualitative: Good	External validation
RISK1 (combined clinical-laboratory indicator model) and RISK2 (laboratory indicator-only model)	Xiaodong Tang et al.	5	(22), (7), (23), (34), (35)	Postoperative Day 1	Risk score calculator	RISK1 model AUC = 0.937; RISK2 model AUC = 0.911	Not reported	Internal validation
Risk-prediction model for anastomotic leakage	Jinrui Wang et al.	5	(49), (24), (37), (32), (36)	Preoperative and intraoperative	Web App	Derivation cohort AUC = 0.750; Validation cohort AUC = 0.723	Brier score = 0.049/0.055, calibration slope = 0.888, calibration intercept = −0.109	Both internal and external validation
LASSO-logistic model	Wenxiang Ma et al.	4	(25), (50), (51), (22)	Based on data from postoperative days 1–3	Integration into electronic health record (EHR) system	Validation cohort: AUC = 0.871; Training cohort: AUC = 0.925	Qualitative: Local deviation	Both internal and external validation
Nomogram for anastomotic leak	R.-H. Tu et al.	3	(49), (42), (26)	Preoperative	Nomogram	0.675	Qualitative: Good	Internal validation
AScore-POD3 based nomogram	Jinyao Shi et al.	6	(11), (12), (13), (14), (15), (16)	Postoperative Day 3	AScore system; Nomogram	Main cohort: 0.93, Validation cohort: 0.82	Qualitative: Good	Both internal and external validation
Support vector machine (SVM) model	Xuanyu Liu et al.	3	(7), (22), (8), (9), (23)	Postoperative Day 1 (POD1), Day 4 (POD4), Day 7 (POD7)	Risk probability output based on feature set and algorithm	0.89	Qualitative: Good	Internal validation
Risk stratification chart and composite model	Ferdinando Carlo Maria Cananzi et al.	2	(10), (7)	Postoperative Day 1 to Day 7	Risk stratification chart	For AL prediction: - CRP at POD7 AUC = 0.772; PCT at POD7 AUC = 0.763	Not reported	Internal validation
Nomogram	Boqi Xu et al.	5	(52), (46), (38), (39), (56)	Preoperative and intraoperative	Nomogram	0.956	Qualitative: Good	Internal validation
Predictive Nomogram	Yi Liao et al.	7	(27), (28), (29), (35), (34), (40), (57)	Preoperative	Nomogram	Development cohort: 0.763; Validation cohort: 0.761	Hosmer–Lemeshow test: *p* = 0.80/*p* = 0.06	Both internal and external validation
LASSO regression risk model	Tiehua Zhao et al.	2	(1), (23)	Postoperative Day 3	Risk score formula	0.854	Qualitative: Good	Internal validation

(1) Diabetes, (2) No Diabetes, (3) Systemic Inflammation Response Index (SIRI) ≥ 1.18, (4) C-reactive protein (CRP) cutoff 113.00 mg/L, (5) Procalcitonin (PCT) cutoff 0.841 ng/mL, (6) White blood cell (WBC) count cutoff 11.56 × 10^9^/L, (7) CRP, (8) Neutrophil-to-Lymphocyte Ratio (NLR), (9) Systemic Immune-Inflammation Index (SII), (10) PCT, (11) Interleukin-1β (IL-1β), (12) Interleukin-6 (IL-6), (13) Interleukin-10 (IL-10), (14) Tumor Necrosis Factor-α (TNF-α), (15) Matrix Metalloproteinase-2 (MMP2), (16) Matrix Metalloproteinase-9 (MMP9), (17) Prognostic Nutritional Index (PNI) ≥ 37, (18) Preoperative Albumin (ALB) level <30 g/L, (19) Hypoalbuminemia < 35 g/L, (20) Preoperative nutritional risk (NRS2002 ≥ 3), (21) Preoperative ALB ≤33.6 g/L, (22) ALB, (23) PNI, (24) Preoperative ALB <35 g/L, (25) ALB on postoperative days 1–3, (26) Malnutrition, (27) NRS-2002 score, (28) Subcutaneous Fat/Muscle Area Index (SFMAI), (29) Visceral-to-Subcutaneous Fat Ratio (VSR), (30) Operation duration ≥303.5 min, (31) Intraoperative blood loss >400 mL, (32) Operation time (≥240 min), (33) Anastomosis method (end-to-side), (34) Operation time, (35) Intraoperative bleeding, (36) Intraoperative blood loss ≥90 mL, (37) Resection extent, (38) Anastomosis type, (39) Blood loss, (40) Reconstruction type, (41) Hemoglobin, (42) Hemoglobin ≤8.0 g/dL, (43) Age ≥60 years, (44) BMI >24 kg/m^2^, (45) History of alcohol consumption, (46) BMI, (47) Brinkman index, (48) American Society of Anesthesiologists Physical Status Classification (ASA score), (49) Age ≥65 years, (50) Age group, (51) History of abdominal surgery, (52) Smoking history, (53) Tumor located in the middle/lower part, (54) Tumor size, (55) Tumor obstruction, (56) Distance from the tumor upper edge, (57) Lauren classification, (58) Pulmonary insufficiency, (59) Pulmonary dysfunction, (60) Hypertension, (61) Anemia.

### 3.3 Quality assessment of the included studies

Detailed quality assessments are provided in the Supplementary materials. The PROBAST was applied to evaluate the risk of bias and applicability of the 18 included models. All 18 studies were judged as having low concern for applicability, indicating that the participants, predictors, and outcome definitions were highly aligned with clinical practice and that no substantial applicability issues were present. With the exception of Shengli Shao, whose model was rated as having an overall low risk of bias, all other studies were rated as having an overall high risk of bias. Unclear ratings in the predictor and outcome domains primarily arose because it was not explicitly stated whether predictor assessment was blinded to outcome data or whether outcome determination was blinded to predictor information. In the analysis domain, all but two studies were at high risk of bias due to an insufficient number of events. The quality assessment of the included studies is presented in Table 4.

1. The risk of bias assessment presented in Table 2 was conducted strictly in accordance with the Prediction model Risk Of Bias ASsessment Tool (PROBAST) developed by Karel G. M. Moons. The latest version of the PROBAST checklist, supporting documentation, and completed examples were downloaded from www.probast.org.
2. In the risk of bias assessment, 94.4% of the studies were rated as having a high risk in the analysis domain; for overall applicability, 100% of the studies were rated as having a low risk.
3. Shao et al. (15, 26) involve the same author, the same prediction model, and the same predictors; the 2025 study represents an external validation of the 2021 study.

**Table 4:** Quality assessment of the included studies.

Included study	Risk of bias					Applicability			
	Participants	Predictors	Outcome	Analysis	Overall	Participants	Predictors	Outcome	Overall
Wang et al. (14)	Low	Low	Unclear	High	High	Low	Low	Low	Low
Shao et al. (2021) (15)	Low	Unclear	Unclear	High	High	Low	Low	Low	Low
Tang et al. (16)	High	Unclear	Unclear	High	High	Low	Low	Low	Low
Wang et al. (17)	Low	Low	Low	High	High	Low	Low	Low	Low
Ma et al. (18)	Low	Unclear	Unclear	High	High	Low	Low	Low	Low
Tu et al. (19)	Low	Unclear	Unclear	High	High	Low	Low	Low	Low
Shi et al. (20)	Low	Unclear	Unclear	High	High	Low	Low	Low	Low
Liu et al. (21)	Low	Low	Low	High	High	Low	Low	Low	Low
Cananzi et al. (22)	Low	Unclear	Low	High	High	Low	Low	Low	Low
Xu et al. (23)	High	Low	Unclear	High	High	Low	Low	Low	Low
Liao et al. (24)	Low	Unclear	Unclear	High	High	Low	Low	Low	Low
Zhao et al.(25)	High	Unclear	Unclear	High	High	Low	Low	Low	Low
Shao et al. (26)	Low	Low	Low	Low	Low	Low	Low	Low	Low
Man et al. (9)	Low	Unclear	Unclear	High	High	Low	Low	Low	Low
Liu et al. (10)	Low	High	High	High	High	Low	Low	Low	Low
Xia et al. (11)	Low	Low	Low	High	High	Low	Low	Low	Low
Deng et al. (12)	Low	Unclear	Unclear	High	High	Low	Low	Low	Low
Zhang et al. (13)	Low	Low	Unclear	High	High	Low	Low	Low	Low

### 3.4 Meta-analysis results

#### 3.4.1 Meta-analysis of external validation

**Figure 2:**
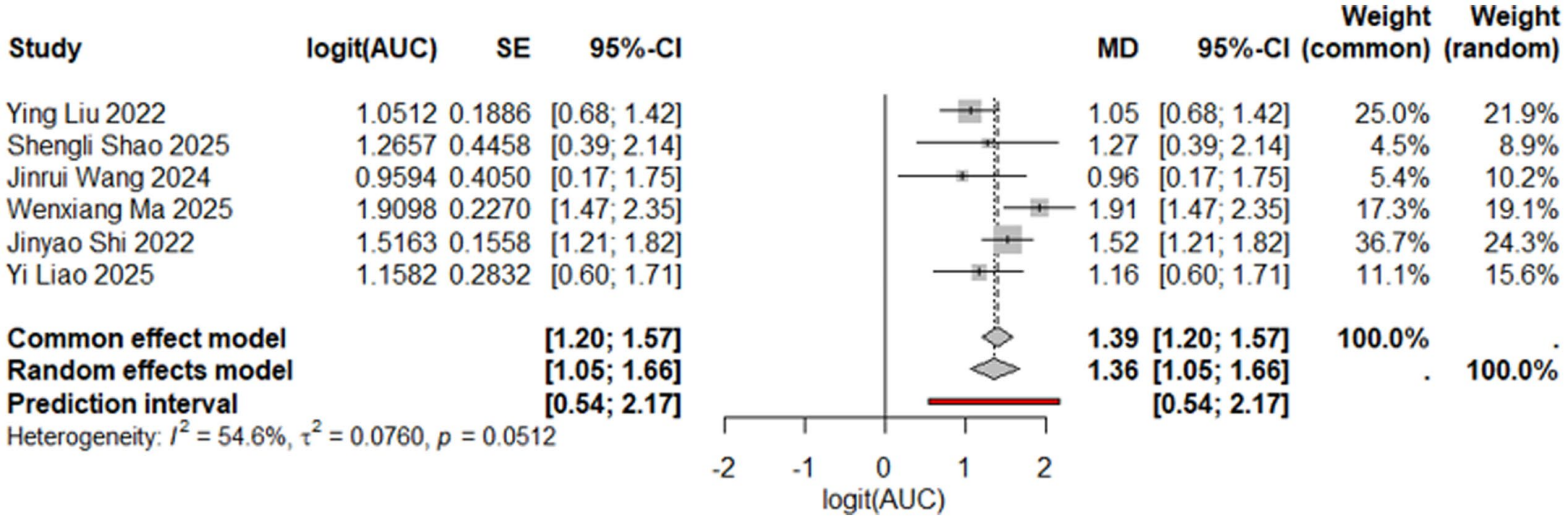
Forest plot of the meta-analysis of external prediction models.

**Figure 3:**
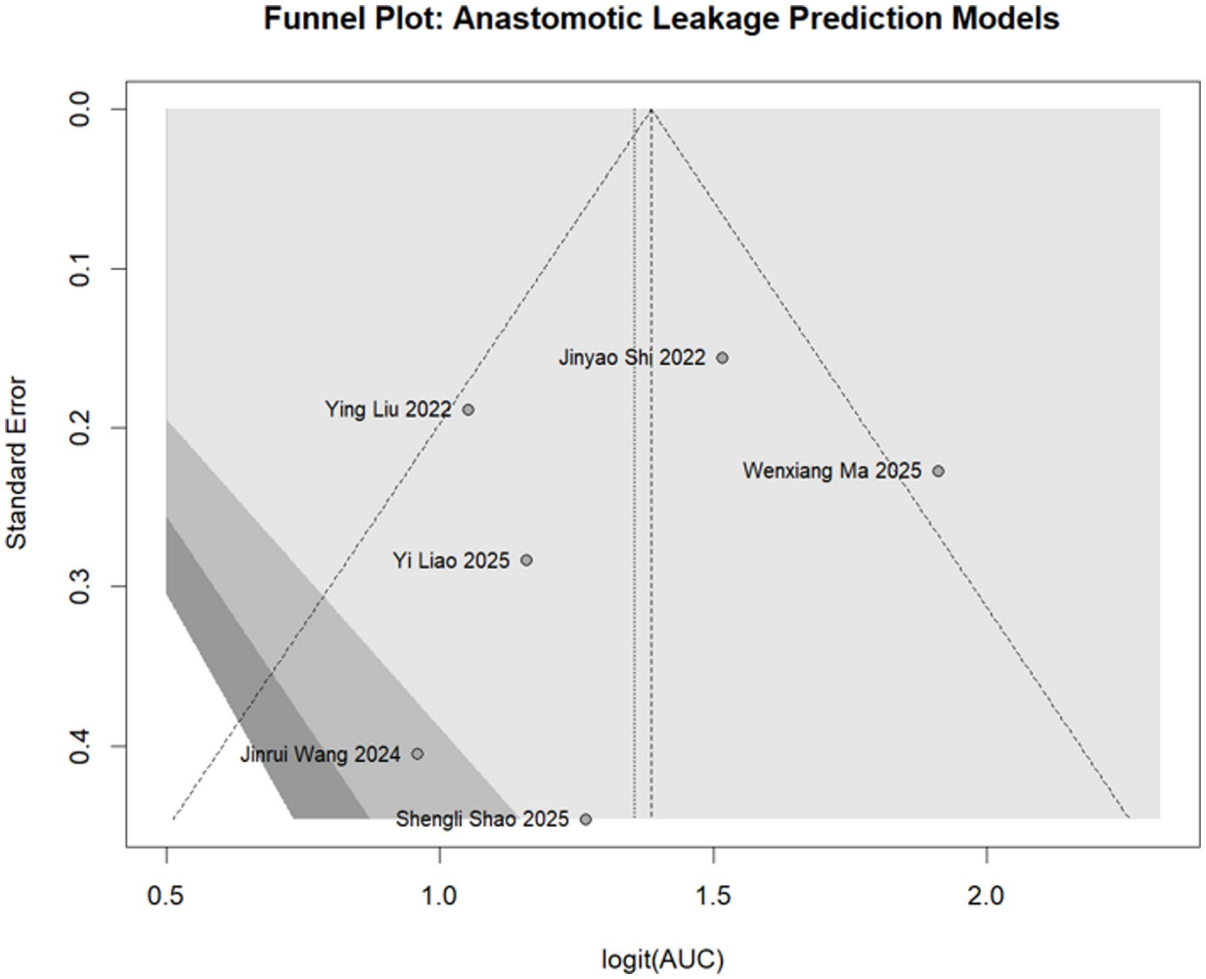
Funnel plot.

To ensure the reliability and stability of the pooled estimates from the prediction models, external validation results from one study that performed only external validation and from five studies that performed both internal and external validation were extracted separately and quantitatively synthesized to evaluate the robustness of the existing external validation. None of the six external validation studies reported standard errors (SEs). The SE was therefore estimated using the formula SE = (upper limit of the confidence interval – lower limit of the confidence interval)/(2 × 1.96) (27). The resulting estimated SEs were 0.0362 for Liu et al. (10), 0.0811 for Wang et al. (17), 0.0255 for Ma et al. (18), 0.0230 for Shi et al. (20), and 0.0515 for Liao et al. (24). Shao et al. (15, 26) did not report a confidence interval; therefore, the 95% confidence interval was approximated as (0.630, 0.930) using the formula of Hanley and McNeil (28) based on the information provided in the article, yielding an estimated SE of 0.0765. It should be noted that these indirect estimations may introduce bias into the pooled effect size, as they cannot precisely reflect the precision of each study and may consequently distort weight assignment in the meta-analysis. The AUC, SE, and sample size (N) from the six external validation studies were transformed using a logit transformation, and a meta-analysis was performed together with a funnel plot for publication bias (the results are shown in Figure 2; the funnel plot is shown in Figure 3). The funnel plot did not display a severely asymmetrical pattern that would raise concern. However, given the small number of included studies, this analysis is insufficient to reliably assess publication bias.

**Figure 4:**
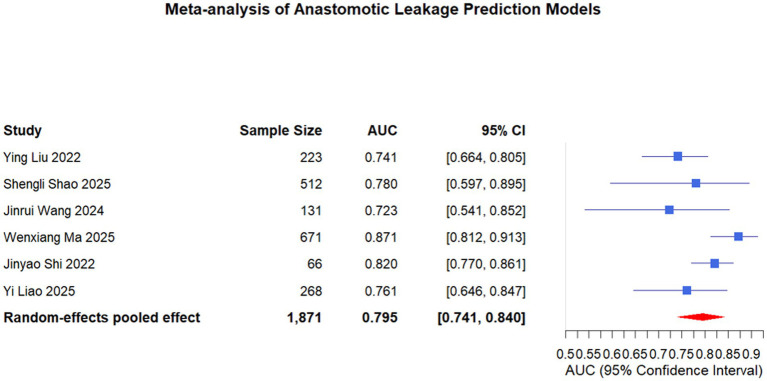
Forest plot of pooled AUC effect sizes.

The meta-analysis showed that the point estimates of logit(AUC) ranged from 0.9594 to 1.9098. Comparison of weight changes and SEs across the studies revealed that Shi et al. (20) was the most precise study and carried the largest weight, while Shao et al. (15, 26) had the lowest precision. Ma et al. (18)showed the highest effect size, while Wang et al. (14, 17) showed the lowest effect size. In the fixed effects model, the 95% CI was [1.20; 1.57] and the logit(AUC) was 1.39 (AUC ≈ 0.8). In the random effects model, the 95% CI was [1.05; 1.66] and the logit(AUC) was 1.36 (AUC ≈ 0.8). The lower bounds of the 95% confidence intervals for both models were greater than zero, indicating that the models’ predictive performance was statistically significant (i.e., the AUC was significantly greater than 0.5), regardless of whether heterogeneity was considered. The prediction interval for the effect size of future similar studies was [0.54; 2.17], corresponding to an AUC range of approximately (0.63, 0.90), meaning that there is a 95% chance that the effect size of a future study will fall within this range, which also reflects between-study heterogeneity and uncertainty. In addition, the I^2^ statistic (54.6%) indicated moderate heterogeneity (29); therefore, the random effects model was selected as the primary method (see Figure 4). The pooled AUC from the random effects model was 0.795, indicating moderately good discriminatory performance. With a value close to 0.8 and a 95% CI of 0.741 to 0.840, the average performance in external validation was judged to be favorable. To explore heterogeneity, a leave-one-out analysis was conducted. The results showed that the exclusion of Wenxiang Ma led to the largest decrease in the pooled AUC (−0.019), indicating that this study substantially increased the overall AUC. After its removal, heterogeneity became very low, suggesting that this study was a key source of heterogeneity (see Table 5).

**Table 5:** Leave-one-out analysis.

Excluded study	Pooled AUC	Change	*I*^2^(%)
Liu et al. (2022) (10)	0.81	0.015	0.4
Shao et al. (2025) (26)	0.796	0.001	0.6
Wang et al. (2024) (26)	0.802	0.007	0.6
Ma et al. (2025) (18)	0.776	−0.019	0.1
Shi et al. (2022) (20)	0.786	−0.009	0.6
Liao et al. (2025) (24)	0.8	0.005	0.6

#### 3.4.2 Subgroup analysis

##### 3.4.2.1 Logit-transformed subgroup analysis of internal and external validation models and optimism bias analysis

**Figure 5:**
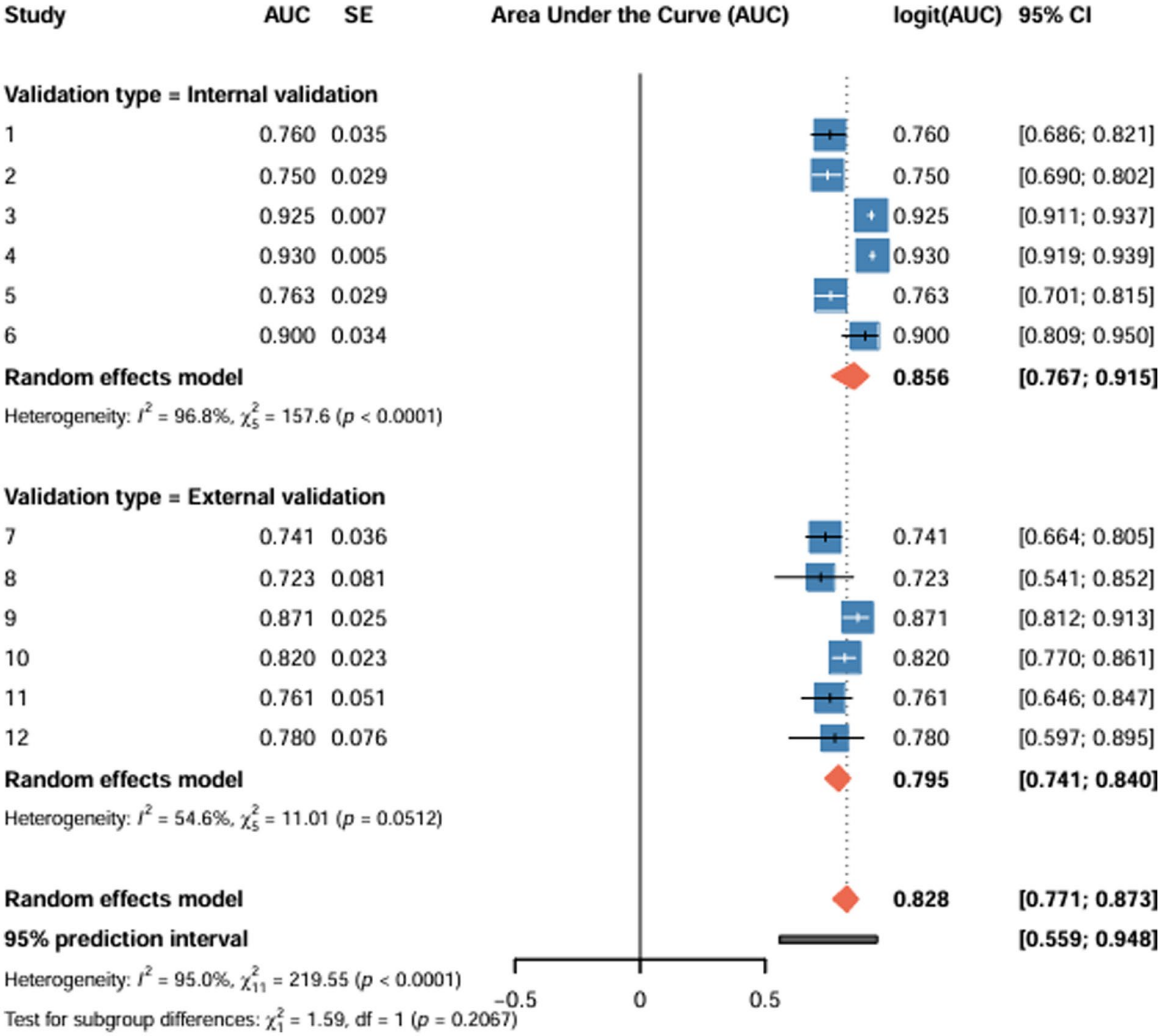
Subgroup analysis of prediction models with internal and external validation.

**Figure 6:**
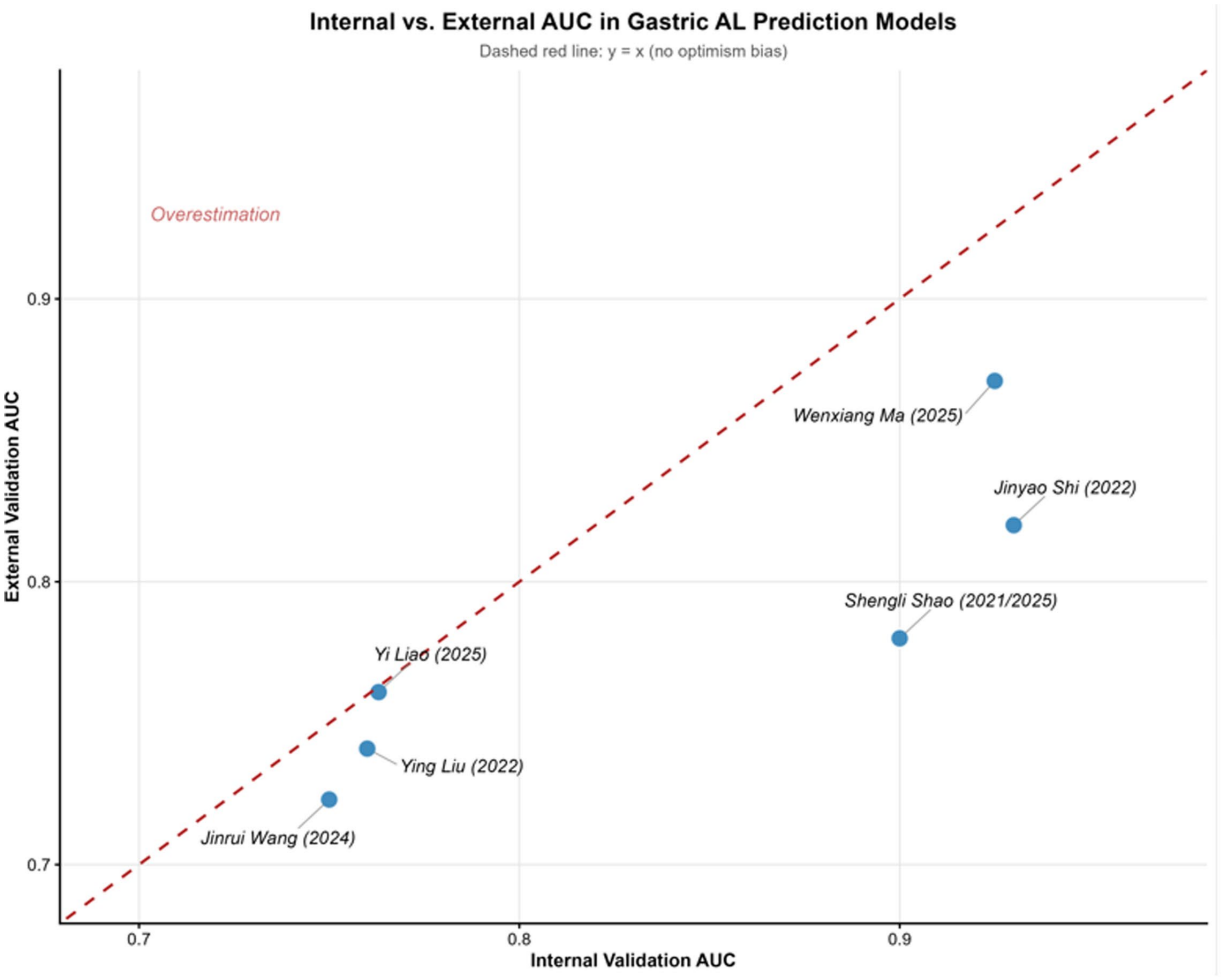
Exploratory scatter plot for optimism bias.

To evaluate and quantify the degree of overfitting in the prediction models, we conducted a subgroup analysis of the six models for which both internal and external validation results were available (see Figure 5). Shao et al. (15, 26) involve the same author and the same model; the 2025 study is a multicenter validation of the 2021 model and was therefore also included. The pooled AUC was 0.856 for internal validation and 0.795 for external validation, yielding an absolute difference of 0.061. This finding suggests that risk prediction models for anastomotic leak after gastric cancer surgery are commonly affected by pronounced optimism bias and overfitting. The logit-transformed AUC decreased by 0.4547 from internal to external validation. Although the between-subgroup difference did not reach statistical significance (*p* = 0.199), the direction and magnitude of the effect were consistent and clear, indicating that the existing models suffer from overfitting and that internal validation substantially overestimates true model performance. To further explore the extent of overfitting, an optimism bias analysis was performed (see Figure 6). The mean absolute difference between internal and external AUCs was 0.055 (range: 0.002–0.12). With the exception of Liao et al. (24), which showed minimal deviation and the best model stability, all models demonstrated higher performance in internal validation than in external validation. The differences for Shi et al. (20) and Shao et al. (15, 26) were relatively large, indicating notable overfitting. The external AUC for Ma et al. (18) was 0.871, the highest among all models.

##### 3.4.2.2 Subgroup analysis by model type

**Figure 7:**
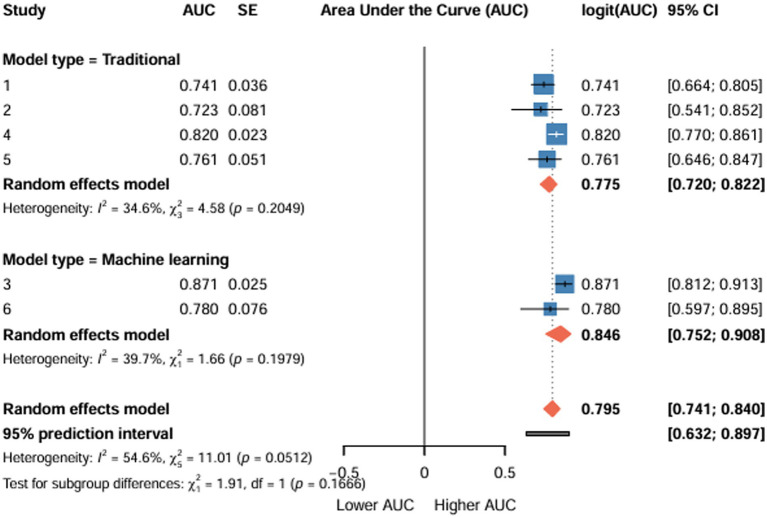
Subgroup analysis by model type.

To examine the influence of model type on predictive performance, the six externally validated studies were stratified by modeling approach into machine learning models (*n* = 2) and traditional regression models (*n* = 4) (see Figure 7). In the random effects model, the pooled AUC was 0.846 (95% CI: 0.752–0.908, I^2^ = 0.4%) for machine learning models and 0.775 (95% CI: 0.720–0.822, I^2^ = 0.3%) for traditional regression models. The difference in the logit-transformed AUC (traditional vs. machine learning) was −0.498 (SE = 0.297), corresponding to an absolute AUC reduction of approximately 0.07. Meta-regression indicated a borderline significant between-subgroup difference (P for interaction = 0.0937). This pattern aligns with findings from previous research; however, because the machine learning subgroup included only two studies, this result should be interpreted as exploratory. Further confirmation through high-quality external validation studies is warranted.

##### 3.4.2.3 Subgroup analysis by timing of data collection

**Figure 8:**
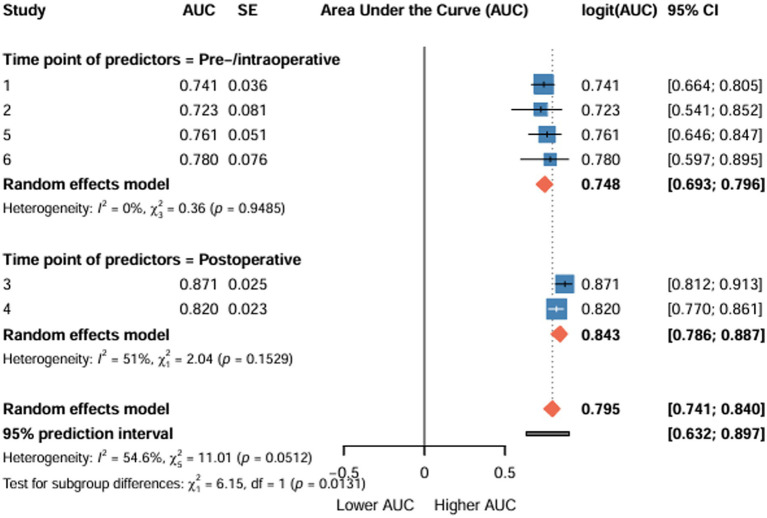
Subgroup analysis by the timing of data collection.

To investigate the impact of the timing of predictor collection on model discriminative performance, the six externally validated studies were divided into two subgroups: Those incorporating postoperative indicators (*n* = 2) and those relying on preoperative/intraoperative indicators (*n* = 4) (see Figure 8). In the random effects model, the pooled AUC was 0.843 for the postoperative dynamic indicator group and 0.748 for the preoperative/intraoperative static indicator group, yielding an absolute difference of 0.095. After a logit transformation, the difference in the pooled logit(AUC) between the two groups was 0.555 (SE = 0.189). This between-subgroup difference reached statistical significance (P for interaction = 0.0034), indicating that the timing of predictor collection is a key determinant of external validation performance and that postoperative dynamic indicators can substantially improve the accuracy of anastomotic leak risk prediction. It should be noted, however, that the postoperative dynamic subgroup included only two studies. Although heterogeneity was very low and the effect size was stable, these findings require further confirmation in studies that incorporate postoperative predictors.

#### 3.4.3 Exploration of sample size and performance in internally validated models

**Figure 9:**
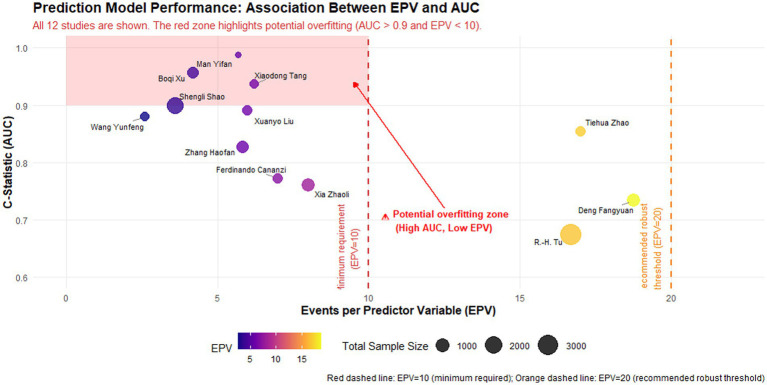
Scatter plots of EPV, AUC, and sample size.

Among the models presented in Tables 4, 12 had not undergone external validation. Their discriminative performance, as measured by the AUC, ranged from 0.675 to 0.988, with a median of 0.854—substantially higher than the pooled AUC of 0.795 from the externally validated models. To further explore the performance of internally validated models, a scatter plot was constructed (Figure 9). In the plot, red/orange points denote models with low events per variable (EPV), indicating a high risk of overfitting, while grey points represent models for which EPV could not be calculated. The red and orange dashed lines correspond to EPV = 10 (the minimum methodological threshold) and EPV = 20 (the ideal target for robustness), respectively. The results showed that models with sample sizes below 500 generally reported higher AUC values, which were often accompanied by low EPV, indicating a heightened risk of overfitting. Among the 11 studies with calculable EPV, only three met the minimum requirement of EPV ≥ 10, and none reached the ideal robustness threshold of EPV ≥ 20. The red-shaded region in the upper-left corner of the plot (AUC > 0.9, EPV < 10) further corroborates this finding. It demonstrates that models developed with small sample sizes are susceptible to unstable performance and optimism bias arising from overfitting or chance (30). Consequently, future prediction model research should shift its focus from attaining high internal validation performance to ensuring adequate sample sizes—particularly high EPV—and to conducting external validation.

#### 3.4.4 Qualitative synthesis

**Figure 10:**
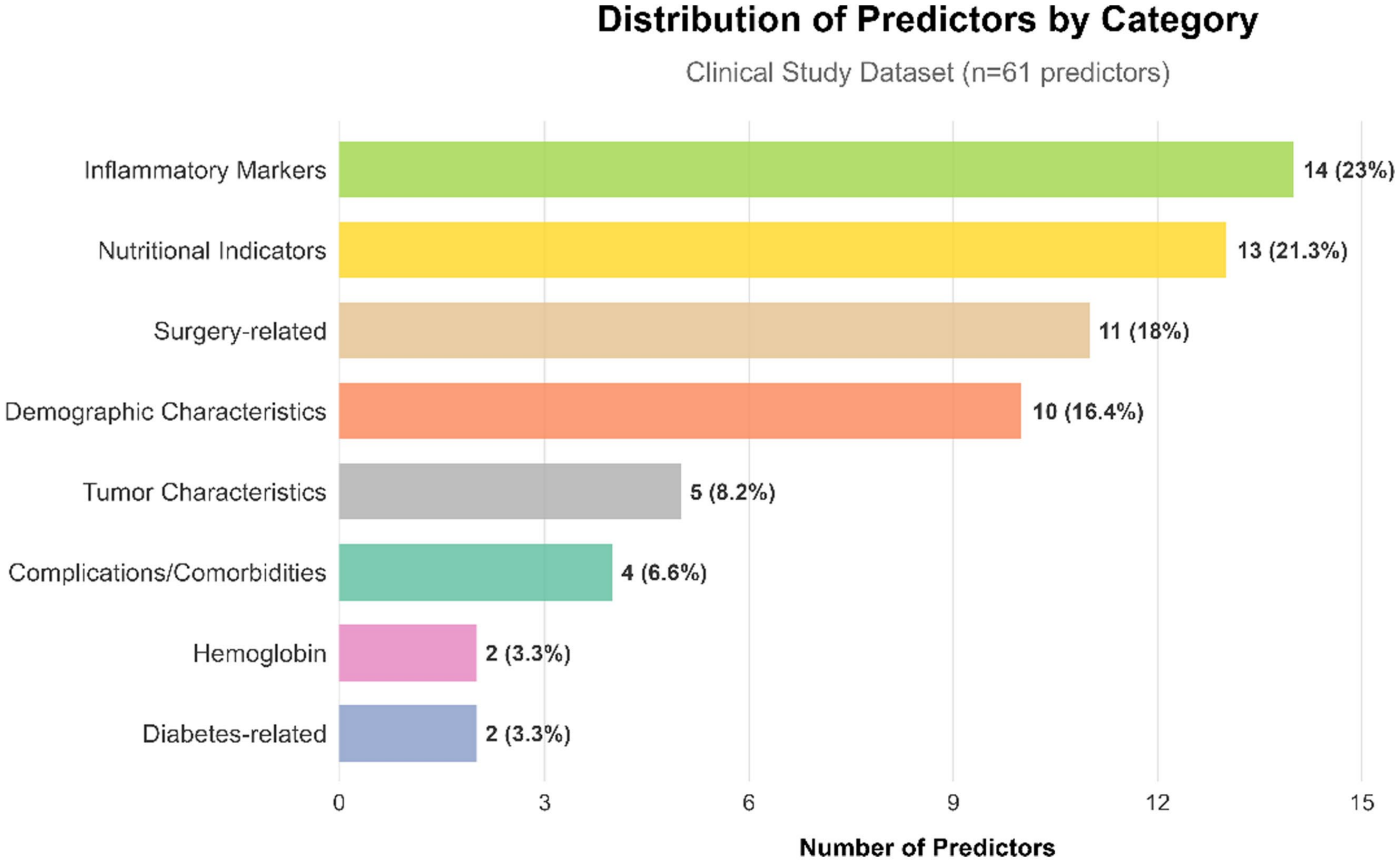
Predictor classification by category.

A qualitative synthesis was performed on the predictors and modeling methods of all included prediction models. As the two studies by Shao et al. (15, 26) share the same model and predictors, only one was retained for this analysis. The 61 unique predictors were classified into eight categories based on their clinical nature (see Figure 10): Diabetes-related factors, inflammatory markers, nutritional markers, surgery-related factors, laboratory parameters, demographic characteristics, tumor characteristics, and comorbidities. Inflammatory markers constituted the largest category (*n* = 14, 23%), underscoring the critical role of the postoperative inflammatory response in predicting complications. Nutritional markers followed closely (*n* = 13, 21.3%), indicating that nutritional status directly influences postoperative recovery and immune function. Surgery-related factors ranked third (*n* = 11, 18%).

The most frequently reported clinical condition was diabetes (including diabetes and history of diabetes), which appeared eight times, suggesting that patients with diabetes are at elevated risk of anastomotic leak after gastric cancer surgery and should be monitored closely. Other predictors reported three or more times included albumin (5 times), operative time (4 times), BMI (3 times), C-reactive protein (CRP) (3 times), and prognostic nutritional index (PNI) (3 times). Most studies lacked information on odds ratios, 95% confidence intervals, and *p*-values for individual predictors, precluding pooled effect size estimation.

Among the 61 predictors, 10 had been validated only internally, including absence of diabetes, tumor located in the middle/lower third of the stomach, systemic inflammation response index (SIRI), PNI, malnutrition, neutrophil-to-lymphocyte ratio (NLR), systemic immune-inflammation index (SII), procalcitonin (PCT), smoking history, and distance from the upper tumor margin. A total of two predictors—white blood cell (WBC) count cutoff of 11.56 × 10^9^/L on postoperative day 5 and history of alcohol consumption—had not undergone any validation. Although these predictors remain insufficiently validated, they possess physiological and clinical significance. Future research should systematically explore and validate these promising novel predictors and investigate new hypotheses.

**Figure 11:**
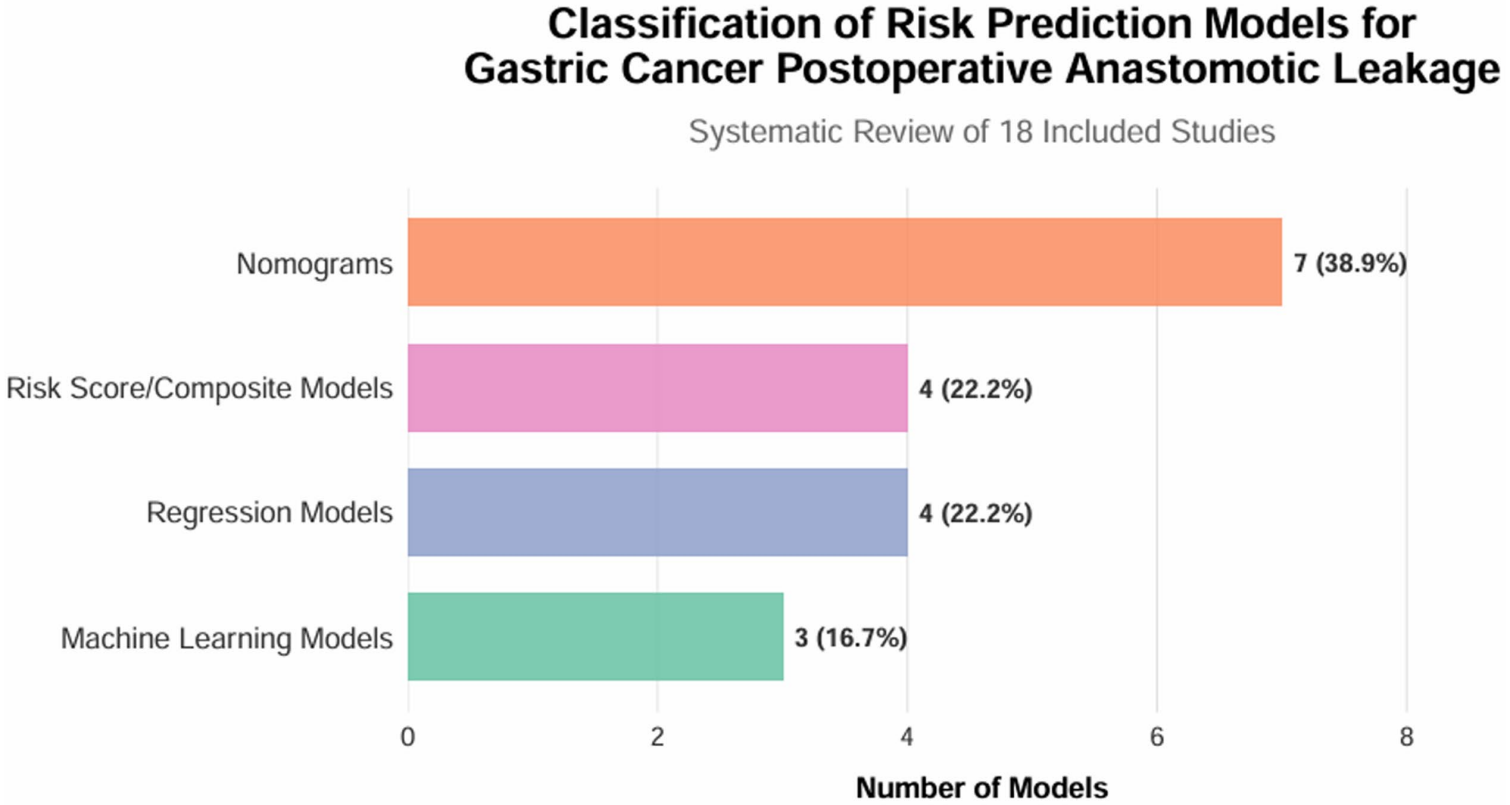
Classification of prediction models.

With regard to model presentation (see Figure 11), nomograms were the most commonly used format (38.9%), likely due to their effective visualization and ease of clinical application. Regression models and risk scores/composite models were tied for second place (22.2% each), while machine learning models were less frequently used (16.7%).

## 4 Discussion

### 4.1 Clinical challenge of AL and the need for risk prediction

Anastomotic leak (AL) is one of the most serious complications following gastric cancer surgery (31). It is not only a leading cause of reoperation, intensive care unit admission, and elevated 30-day mortality but also profoundly compromises long-term survival (32–34). Despite continuous advances in surgical techniques and materials (35–38), AL remains a major postoperative complication that affects prognosis. Its pathogenesis is complex, and clinical experience alone is insufficient to achieve accurate early warning (39–41). The present study, therefore, provides a comprehensive evaluation of external validation performance, sources of heterogeneity, risk of overfitting, and the spectrum of predictors in AL prediction models after gastric cancer surgery, with the aim of informing future model development and clinical translation.

### 4.2 Summary of key findings

This systematic review and meta-analysis evaluated risk prediction models for AL after gastric cancer surgery, including 18 studies encompassing 15,783 patients. The pooled AUC from external validation was 0.795 (95% CI: 0.741–0.840), indicating moderately good overall discriminative ability, with moderate heterogeneity (I^2^ = 54.6%). It is worth noting that the meta-analysis included only six datasets from externally validated prediction models, and these models varied substantially in terms of predictor composition, timing of predictor collection, modeling methods, and validation strategies. Although the random effects model partly accommodates this variability, the pooled AUC should be regarded as an exploratory estimate, and local validation is required when applying these models to new settings. The I^2^ of 54.6% was largely attributable to Ma et al. (18); after the exclusion of this study, the I^2^ dropped to 0.1%. This study employed a LASSO-logistic model, which demonstrated outstanding performance and exerted a considerable influence on the pooled effect size.

In the model-type subgroup analysis, the pooled AUC for machine learning models (0.846) was higher than that for traditional regression models (0.775), with a difference of approximately 0.07 (*p* = 0.094). This difference was insufficient to support a definitive advantage of machine learning over traditional regression models. Moreover, the machine learning subgroup included only two externally validated studies. Thus, the apparent performance advantage of machine learning models may partially stem from overfitting, small sample size effects, or insufficient validation, rather than from inherent methodological superiority, and should be interpreted cautiously.

Notably, the timing of predictor collection was the only subgroup factor that reached statistical significance (*p* = 0.0034). The pooled AUC for models incorporating postoperative dynamic indicators was 0.843 (95% CI: 0.791–0.884), which was significantly higher than that for models relying on preoperative/intraoperative static indicators (0.748, 95% CI: 0.690–0.798), with a significant between-subgroup difference (P for interaction = 0.0034). This suggests that postoperative dynamic monitoring parameters (e.g., CRP, albumin, and drain fluid inflammatory cytokines on postoperative days 1–3) can improve predictive accuracy for AL risk. Although the postoperative dynamic subgroup included only two studies, limiting definitive conclusions, heterogeneity was extremely low and the effect size was stable, indicating that future model development may benefit from a greater focus on postoperative dynamic indicators.

Furthermore, exploratory analysis of internally validated models revealed that studies with small sample sizes and low events per variable (EPV) ratios were more likely to report higher AUC values, indicating that overfitting may be a common problem in current model research. Among the 11 studies with calculable EPV, only three met the minimum methodological requirement of EPV ≥ 10, and none reached the ideal robustness target of EPV ≥ 20, suggesting that model performance may be influenced by insufficient sample size and chance effects. Therefore, future model development should place greater emphasis on adequate sample size design (particularly increasing EPV) and robust external validation, rather than focusing solely on high internal validation performance.

We also found that calibration was frequently reported only through subjective descriptions of calibration plots, with a notable absence of quantitative metrics such as the calibration intercept, calibration slope, and Brier score; some studies did not report any calibration results. Models with poor calibration may overestimate or underestimate true risk even when the AUC is high, thereby compromising the reliability of clinical decision-making. Moreover, among the externally validated studies, only a few models reported both discrimination and calibration metrics, indicating that the current development of AL prediction models for gastric cancer surgery still places disproportionate emphasis on discriminative ability while paying insufficient attention to the accuracy of predicted probabilities. Future studies must adhere to the TRIPOD statement and PROBAST criteria, requiring comprehensive reporting of both discrimination and calibration, as well as rigorous external calibration validation, to enhance real-world clinical utility.

### 4.3 Clinical applicability and the implementation gap

Most of the current models exhibit moderate-to-good discriminative ability, yet their clinical value requires further evaluation. Nomograms and web-based applications were the most common model presentation formats. However, model visualization does not necessarily translate into clinical implementability. Some high-performing models rely on postoperative dynamic indicators; although such indicators can improve predictive accuracy, their clinical utility depends on whether they are obtained early enough to meaningfully influence clinical decision-making. For example, early postoperative risk alerts could enable clinicians to adjust monitoring intensity, optimize anti-infective regimens, or prepare for timely reoperation—interventions that may reduce the severity and mortality associated with anastomotic leak. However, if high-risk identification occurs only after the leak has already formed or become clinically evident, the incremental value may be limited. In addition, although some machine learning models have been deployed as web-based tools or electronic health record decision-support systems, implementation studies and impact analyses are currently lacking. No study has yet examined whether using these models genuinely alters clinical decisions or improves patient outcomes. Future research should encompass prospective, pragmatic studies that focus on assessing the real-world impact of these models on clinical decision-making and patient-important outcomes (e.g., severity of anastomotic leak, reoperation rate, length of hospital stay, and mortality).

### 4.4 Methodological considerations and limitations

The models included in this review exhibit substantial clinical and methodological heterogeneity; thus, the pooled AUC is best regarded as an approximate description of the overall discriminative performance of current AL risk prediction models for gastric cancer surgery. Importantly, one of the most critical limitations of this study is that only six studies reported external validation. This small number fundamentally limits the robustness of the subgroup analyses and meta-regression results, and the subgroup findings should be viewed as exploratory rather than conclusive. Methodologically, the six externally validated original studies did not directly report standard errors (SEs), and one study did not provide a confidence interval (CI). SEs were estimated using the standard error formula and the Hanley and McNeil (18) formula. Although these methods are statistically justifiable when primary variance data are missing, they may still affect the weighting of studies and the estimation of the pooled effect size, thereby increasing uncertainty in the meta-analytic results. Interpretation of heterogeneity should consider the clinical context; clinical translation and decision support require consideration of a model’s AUC, sensitivity, specificity, and decision curve analysis to determine the optimal risk threshold (42). Moreover, the included studies were predominantly retrospective in design (15/18), which may introduce selection bias and measurement bias. The 18 articles involved 23 Chinese medical institutions and three Italian medical institutions. This geographic bias severely limits the generalizability of our findings to other global populations, healthcare systems, and surgical practices. Patient characteristics, perioperative management, surgical techniques, and definitions of anastomotic leak may vary considerably across regions. Consequently, robust evidence is still lacking regarding the predictive performance and stability of current models in non-Chinese populations, which may be affected by region-specific clinical practice patterns and center effects. Future research should prioritize multinational, multicenter external validation to improve international applicability and transportability. In addition, because the literature search was conducted up to 25 December 2025, this review will need to be updated periodically.

## 5 Conclusion

This systematic review evaluated the quality and predictive performance of risk prediction models for anastomotic leak after gastric cancer surgery. Existing models demonstrate moderately good overall discriminative ability in external validation, but they are commonly affected by overfitting risk and methodological bias. Subgroup analysis suggests that models incorporating postoperative dynamic indicators may achieve better predictive performance, although this finding requires further confirmation. Current model development is still hampered by insufficient external validation, inadequate reporting of calibration, and geographic concentration. Future research should prioritize large-sample, prospective, multicenter, and cross-regional external validation and should strengthen implementation and clinical utility studies to enhance the reliability and generalizability of prediction models.

## Data Availability

The original contributions presented in the study are included in the article/Supplementary material, further inquiries can be directed to the corresponding authors.

## Supplementary material

Supplementary materials Detailed search strategies and PROBAST assessment for each study.

## References

[1] Bray F Laversanne M Sung H Ferlay J Siegel RL Soerjomataram I . Global cancer statistics 2022: GLOBOCAN estimates of incidence and mortality worldwide for 36 cancers in 185 countries. CA Cancer J Clin. (2024) 74:229–63. doi: 10.3322/caac.21834, 38572751

[2] Lordick F Carneiro F Cascinu S Fleitas T Haustermans K Piessen G . Gastric cancer: ESMO clinical practice guideline for diagnosis, treatment and follow-up. Ann Oncol. (2022) 33:1005–20. doi: 10.1016/j.annonc.2022.07.004 35914639

[3] Russell MC Mansfield PF. Surgical approaches to gastric cancer. J Surg Oncol. (2013) 107:250–8. doi: 10.1002/jso.23180 22674546

[4] Du N Ma WX Xie JJ Yang Y Yu YJ. Research progress on risk prediction models for anastomotic leak after gastric cancer resection. Chin J Bases Clin Gen Surg. (2025) 32:916–24. doi: 10.7507/1007-9424.202504071 (in Chinese)

[5] Moons KG Moons KGM de Groot JAH Bouwmeester W Vergouwe Y Mallett S . Critical appraisal and data extraction for systematic reviews of prediction modelling studies: the CHARMS checklist. PLoS Med. (2014) 11:e1001744. doi: 10.1371/journal.pmed.1001744, 25314315 PMC4196729

[6] Moons KGM Wolff RF Riley RD Whiting PF Westwood M Collins GS . PROBAST: a tool to assess risk of Bias and applicability of prediction model studies: explanation and elaboration. Ann Intern Med. (2019) 170:W1–w33. doi: 10.7326/M18-1377, 30596876

[7] Debray TP Damen JA Snell KI Ensor J Hooft L Reitsma JB . A guide to systematic review and meta-analysis of prediction model performance. BMJ. (2017) 356:i6460. doi: 10.1136/bmj.i6460 28057641

[8] Cumpston M Li T Page MJ Chandler J Welch VA Higgins JP . Updated guidance for trusted systematic reviews: a new edition of the Cochrane handbook for systematic reviews of interventions. Cochrane Database Syst Rev. (2019) 10:Ed000142. doi: 10.1002/14651858.ED000142 31643080 PMC10284251

[9] Man YF. Significance of White Blood Cell Count, C-Reactive Protein and Procalcitonin in Predicting Anastomotic Leak after Radical Gastrectomy for Gastric Cancer. Qingdao, China: Qingdao University (2021).

[10] Liu Y Gong XD Wang XP. Establishment and validation of a nomogram prediction model for anastomotic leak after radical total gastrectomy. Chin Foreign Med Res. (2022) 20:179–84. doi: 10.14033/j.cnki.cfmr.2022.26.047 (in Chinese)

[11] Xia ZL. Evaluation and Analysis of Risk Factors for Anastomotic Leak after Total Gastrectomy for Esophagogastric Junction Tumors. Shanghai, China: Naval Medical University (2021).

[12] Deng FY Huang J Peng HX Yin CM Yang BR. Risk factor analysis of anastomotic leak after radical gastrectomy for gastric cancer and establishment of a risk prediction scoring model. Chin J Dig Surg. (2019) 18:259–63. doi: 10.3760/cma.j.issn.1673-9752.2019.03.011 (in Chinese)

[13] Zhang HF. Risk Factor Analysis and Prediction Model Construction for Anastomotic Leak after Gastric Cancer Surgery. Taiyuan, China: Shanxi Medical University (2024) (in Chinese).

[14] Wang YF Guo ZQ Han JX Gao LN Liu YM Jia K . Analysis of risk factors for esophagojejunal anastomotic leakage after total gastrectomy based on Bayesian network model. Front Med (Lausanne). (2025) 12:1632214. doi: 10.3389/fmed.2025.1632214, 40837566 PMC12361184

[15] Shao SL Liu L Zhao YF Mu L Lu QY. Application of machine learning for predicting anastomotic leakage in patients with gastric adenocarcinoma who received Total or proximal gastrectomy. J Personal Med. (2021) 11:748. doi: 10.3390/jpm11080748 PMC8400241 34442391

[16] Tang XD Jin T Zhang XH Tang XL Ding XL. Clinical value of prognostic nutritional index combined with C-reactive protein and albumin in early prediction of anastomotic leakage after radical gastric cancer surgery. Am J Transl Res. (2024) 16:3081–9. doi: 10.62347/LDOZ1986, 39114734 PMC11301467

[17] Wang JR Liu XL Pan HY Xu YH Wu MZ Li XP . Construction and validation of a risk-prediction model for anastomotic leakage after radical gastrectomy: a cohort study in China. Laparosc Endosc Robot Surg. (2024) 7:34–43. doi: 10.1016/j.lers.2024.02.003

[18] Ma W Zhao S du N Xie J Yang Y Liu J . Development of a machine learning-based predictive model for anastomotic leakage following gastric Cancer surgery. Am Surg. (2025) 92:1189–205. doi: 10.1177/00031348251391850 41126480

[19] Tu RH Lin JX Zheng CH Li P Xie JW Wang JB . Development of a nomogram for predicting the risk of anastomotic leakage after a gastrectomy for gastric cancer. Eur J Surg Oncol. (2017) 43:485–92. doi: 10.1016/j.ejso.2016.11.022, 28041649

[20] Shi JY Wu ZQ Wu XL Shan F Zhang Y Ying XJ . Early diagnosis of anastomotic leakage after gastric Cancer surgery via analysis of inflammatory factors in abdominal drainage. Ann Surg Oncol. (2022) 29:1230–41. doi: 10.1245/s10434-021-10763-y, 34550478

[21] Liu XY Lei S Wei Q Wang YZ Liang HB Chen L . Machine learning-based correlation study between perioperative Immunonutritional index and postoperative anastomotic leakage in patients with gastric Cancer. Int J Med Sci. (2022) 19:1173–83. doi: 10.7150/ijms.72195, 35919820 PMC9339417

[22] Cananzi FCM Biondi A Agnes A Ruspi L Sicoli F de Pascale S . Optimal predictors of postoperative complications after gastrectomy: results from the Procalcitonin and C-reactive protein for the early diagnosis of anastomotic leakage in Esophagogastric surgery (PEDALES) study. J Gastrointest Surg. (2023) 27:478–88. doi: 10.1007/s11605-022-05547-y, 36509900

[23] Xu BQ Zhang F Peng Y Tong S. Predicting esophagojejunal anastomotic leakage in gastric cancer patients after total gastrectomy: development and assessment of a new predictive nomogram. Asian J Surg. (2024) 47:528–30. doi: 10.1016/j.asjsur.2023.09.093, 37775374

[24] Liao Y Lv L Lin F Li W Ji X Liu Z . Predictive value and model construction of preoperative nutritional indexes for postoperative leakage in gastric cancer. Nutrition. (2025) 131:112630. doi: 10.1016/j.nut.2024.112630, 39608342

[25] Zhao TH Li L Wang Y Xie WQ Liu QG . Prognostic nutritional index combined with carcinoembryonic antigen and carbohydrate antigen 242 for early prediction of anastomotic leakage after radical gastrectomy for gastric cancer. Am J Transl Res. (2023) 15:4668–77. 37560224 PMC10408503

[26] Shao SL Li YQ Cheng HR Chen C Zeng Y Huang WJ . Prospective multicenter validation of a machine learning model for predicting anastomotic leakage in patients with gastric adenocarcinoma undergoing total or proximal gastrectomy. Int J Surg. (2025) 111:8027–36. doi: 10.1097/JS9.0000000000003025, 40696942 PMC12626478

[27] Altman DG Bland JM. How to obtain the confidence interval from a P value. BMJ. (2011) 343:d2090. doi: 10.1136/bmj.d2090 21824904

[28] Hanley JA McNeil BJ. The meaning and use of the area under a receiver operating characteristic (ROC) curve. Radiology. (1982) 143:29–36. doi: 10.1148/radiology.143.1.7063747, 7063747

[29] Higgins JP Thompson SG. Quantifying heterogeneity in a meta-analysis. Stat Med. (2002) 21:1539–58. doi: 10.1002/sim.1186 12111919

[30] Riley RD Ensor J Snell KIE Harrell FE Jr Martin GP Reitsma JB . Calculating the sample size required for developing a clinical prediction model. BMJ. (2020) 368:m441. doi: 10.1136/bmj.m441 32188600

[31] Chiarello MM Bianchi V Fransvea P Brisinda G. Endoluminal vacuum-assisted therapy as a treatment for anastomotic leakage in colorectal surgery. World J Gastroenterol. (2022) 28:3747–52. doi: 10.3748/wjg.v28.i28.3747 36161042 PMC9372806

[32] van Hootegem SJM van der Linde M Schneider MA Kim J Berlth F Sugita Y . Impact of postoperative complications on clinical outcomes after gastrectomy for cancer: multicentre study. Br J Surg. (2025) 112:znaf043. doi: 10.1093/bjs/znaf043 40156166 PMC11953074

[33] Aurello P Magistri P D'Angelo F Valabrega S Sirimarco D Tierno SM . Treatment of esophagojejunal anastomosis leakage: a systematic review from the last two decades. Am Surg. (2015) 81:450–3. doi: 10.1177/000313481508100523 25975326

[34] Kamarajah SK Navidi M Griffin SM Phillips AW. Impact of anastomotic leak on long-term survival in patients undergoing gastrectomy for gastric cancer. Br J Surg. (2020) 107:1648–58. doi: 10.1002/bjs.11749, 32533715

[35] Mori M Shuto K Hirano A Kosugi C Narushima K Hosokawa I . A novel parameter identified using Indocyanine green fluorescence angiography may contribute to predicting anastomotic leakage in gastric Cancer surgery. World J Surg. (2020) 44:2699–708. doi: 10.1007/s00268-020-05488-0, 32227275

[36] Grigor EJM Kaaki S Fergusson DA Maziak DE Seely AJE. Interventions to prevent anastomotic leak after esophageal surgery: a systematic review and meta-analysis. BMC Surg. (2021) 21:42. doi: 10.1186/s12893-020-01026-w, 33461529 PMC7814645

[37] Cira K Stocker F Reischl S Obermeier A Friess H Burgkart R . Coating of intestinal anastomoses for prevention of postoperative leakage: a systematic review and Meta-analysis. Front Surg. (2022) 9:882173. doi: 10.3389/fsurg.2022.882173, 35769150 PMC9235828

[38] Aly M O'Brien JW Clark F Kapur S Stearns AT Shaikh I. Does intra-operative flexible endoscopy reduce anastomotic complications following left-sided colonic resections? A systematic review and meta-analysis. Color Dis. (2019) 21:1354–63. doi: 10.1111/codi.14740, 31243879

[39] Huh YJ Lee HJ Kim TH Choi YS Park JH Son YG . Efficacy of assessing intraoperative bowel perfusion with near-infrared camera in laparoscopic gastric Cancer surgery. J Laparoendosc Adv Surg Tech A. (2019) 29:476–83. doi: 10.1089/lap.2018.0263 30589374

[40] Ohi M Saigusa S Toiyama Y Ichikawa T Shimura T Yasuda H . Evaluation of blood flow with Indocyanine green-guided imaging to determine optimal site for gastric conduit anastomosis to prevent anastomotic leak after Esophagectomy. Am Surg. (2017) 83:e197–9. doi: 10.1177/000313481708300607, 28637544

[41] Sierzega M Kolodziejczyk P Kulig J. Impact of anastomotic leakage on long-term survival after total gastrectomy for carcinoma of the stomach. Br J Surg. (2010) 97:1035–42. doi: 10.1002/bjs.7038 20632269

[42] Vickers AJ Elkin EB. Decision curve analysis: a novel method for evaluating prediction models. Med Decis Mak. (2006) 26:565–74. doi: 10.1177/0272989X06295361 PMC2577036 17099194

