# Supplementary material for: Systematic review and meta-analysis of risk prediction models for anastomotic leak after gastric cancer surgery
Source: Front Med (Lausanne). 2026 Jun 12;13:1855091. doi: 10.3389/fmed.2026.1855091 (PMC13303016; doi:10.3389/fmed.2026.1855091)
Supplement: Supplementary materials — Detailed search strategies and PROBAST assessment for each study. [file Data_Sheet_1.PDF]

21 studies from CNKI

（主题：胃肿瘤 + 胃癌 + 胃腺癌）AND（主题：胃切除术 + 胃切除 + 胃癌手术 + 根治性切除 + 外科手术）AND（主题：吻合口漏 + 吻合口瘘 + 消化道瘘 + 吻合口并发症 + 术后漏 + 吻合口 + 漏 + 瘘）AND（主题：预测模型 + 风险预测 + 预测评分 + 风险模型 + 列线图 + 诺莫图 + 预测工具 + 评分系统验证 + 外部验证 + 性能评价 + 区分度 + 校准度）

Wanfang Data: 213 articles

主题:((胃肿瘤 OR 胃癌 OR 胃腺癌) AND (胃切除术 OR 胃切除 OR 胃癌手术 OR 根治性切除 OR 外科手术)) and 主题:(吻合口漏 OR 吻合口瘘 OR 消化道瘘 OR 吻合口并发症 OR 术后漏 OR 吻合口 OR 漏 OR 瘘) and 主题:(预测模型 OR 风险预测 OR 预测评分 OR 风险模型 OR 列线图 OR 诺莫图 OR 预测工具 OR 评分系统 OR 验证 OR 外部验证 OR 性能评价 OR 区分度 OR 校准度)

VIP: 31 articles

((((((((任意字段=胃肿瘤 OR 任意字段=胃癌) OR 任意字段=胃腺癌) AND (((任意字段=胃切除术 OR 任意字段=胃切除) OR 任意字段=胃癌手术OR 根治性切除) OR 任意字段=外科手术))) AND (((((((任意字段=吻合口漏 OR 任意字段=吻合口瘘) OR 任意字段=消化道瘘) OR 任意字段=吻合□并发症) OR 任意字段=术后漏) OR 任意字段=吻合口) OR 任意字段=漏) OR 任意字段=瘘))) AND (((((((((((((任意字段=预测模型 OR 任意字段=风险预测) OR 任意字段=预测评分) OR 任意字段=风险模型) OR 任意字段=列线图) OR 任意字段=诺莫图) OR 任意字段=预测工具) OR 任意字段=评分系统) OR 任意字段=验证) OR 任意字段=外部验证) OR 任意字段=性能评价) OR 任意字段=区分度) OR 任意字段=校准度)))

SinoMed: 56 articles

10	(((#1) AND (#2)) AND ((#3) OR (#4) OR (#5)) AND ((#6) OR (#7)))	56	15:44:18	
7	"验证"[常用字段:智能] OR "外部验证"[常用字段:智能] OR "性能评价"[常用字段:智能] OR "区分度"[常用字段:智能] OR "校准度"[常用字段:智能]	175953	14:28:02	
6	"预测模型"[常用字段:智能] OR "风险预测"[常用字段:智能] OR "预测评分"[常用字段:智能] OR "风险模型"[常用字段:智能] OR "列线图"[常用字段:智能] OR "诺莫图"[常用字段:智能] OR "预测工具"[常用字段:智能] OR "评分系统"[常用字段:智能]	59737	14:26:53	
5	"漏"[常用字段:智能] OR "瘘"[常用字段:智能]	254752	14:23:25	
4	"吻合口"[常用字段:智能]	31218	14:23:01	
3	"吻合口漏"[常用字段:智能] OR "吻合口瘘"[常用字段:智能] OR "消化道瘘"[常用字段:智能] OR "吻合口并发症"[常用字段:智能] OR "术后漏"[常用字段:智能]	19152	14:22:17	
2	"胃切除术"[常用字段:智能] OR "胃切除"[常用字段:智能] OR "胃癌手术"[常用字段:智能] OR "根治性切除"[常用字段:智能] OR "外科手术"[常用字段:智能]	2219378	14:21:07	
1	"胃肿瘤"[常用字段:智能] OR "胃癌"[常用字段:智能] OR "胃腺癌"[常用字段:智能]	134980	14:20:00	

PubMed: 3,377 articles

Search: **(("Stomach Neoplasms"[Mesh] OR "Gastric Cancer"[tiab] OR "Stomach Cancer"[tiab] OR "Gastric Carcinoma"[tiab] OR "Gastrectomy"[Mesh] OR gastrectomy[tiab]) AND ("Anastomotic Leak"[Mesh] OR "Anastomosis, Surgical/adverse effects"[Mesh] OR "Postoperative Complications"[Mesh] OR ((anastomotic[tiab] OR anastomosis[tiab]) AND (leak*[tiab] OR fistula*[tiab] OR failure[tiab] OR complication*[tiab])) OR "AL"[tiab])) AND ("Risk Assessment"[Mesh] OR "Predictive Value of Tests"[Mesh] OR "Models, Statistical"[Mesh] OR "Nomograms"[Mesh] OR predict*[tiab] OR model[tiab] OR models[tiab] OR score[tiab] OR scores[tiab] OR rule[tiab] OR rules[tiab] OR calculator[tiab] OR nomogram*[tiab])**

Web of Science (WOS): 583 articles

(TS=("gastric cancer" OR "stomach cancer" OR "gastric carcinoma" OR gastrectomy)) AND
(TS=("anastomotic leak" OR AL OR ((anastomotic OR anastomosis) NEAR/3 (leak* OR fistula* OR failure OR complication)))) AND
(TS=(predict OR model OR models OR score OR scores OR rule OR rules OR calculator OR nomogram* OR "risk assessment" OR "predictive value"))

https://www.webofscience.com/wos/woscc/summary/1f442120-acbb-4311-8f80-41dc0b18ba38-0193b65c8a/relevance/1

Embase: 709 articles

#16	#13 AND #14 AND #15	709
#15	'prediction'/exp OR 'nomogram'/exp OR 'risk assessment'/exp OR ((predict* NEAR/3 (model OR score OR rule OR calculator OR n	Edit Email alert RSS feed
#14	'anastomosis leakage'/exp OR ((anastomotic NEAR/3 leak*):ti,ab) OR 'al':ti,ab	915,411
#13	'stomach tumor'/exp OR 'gastrectomy'/exp OR 'gastric cancer':ti OR 'stomach cancer':ti OR 'gastric carcinoma':ti	289,632

CINAHL: 7 articles

正在检索: CINAHL Complete （另外 1 个） 基本检索 PICOT

(MH "Stomach Neoplasms+") OR TI ("Gastric Cancer" OR "Stomach Cancer") OR AB ("Gastric Cancer" OR "Stomach Cancer") — 所有字段

AND (MH "Anastomotic Leak+") OR TI "anastomotic leak*" OR AB "anastomotic leak*" — 所有字段

AND (MH "Risk Assessment" OR MH "Models, Statistical") OR TI (predict* N3 (model OR score OR nomogram)) OR AB (predict* N3 (model OR score OR nomogram)) — 所有字段

Cochrane Library: 46 articles

#1 MeSH descriptor: [Stomach Neoplasms] explode all trees 4313

#2 MeSH descriptor: [Adenocarcinoma] explode all trees 11683

#3 (gastric cancer*):ti,ab,kw OR (stomach cancer*):ti,ab,kw OR (gastric carcinoma*):ti,ab,kw OR (gastric adenocarcinoma*):ti,ab,kw OR (gastric neoplasm*):ti,ab,kw 13379

#4 (stomach neoplasm*):ti,ab,kw 5764

#5 #1 OR #2 OR #3 OR #4 24243

#6 MeSH descriptor: [Gastrectomy] explode all trees1839

#7 (gastrectom*):ti,ab,kw OR (stomach resection*):ti,ab,kw OR (gastric resection*):ti,ab,kw OR (gastric surgery):ti,ab,kw OR (radical gastrectomy):ti,ab,kw 13944

#8 (total gastrectomy):ti,ab,kw OR (distal gastrectomy):ti,ab,kw OR (D2 resection):ti,ab,kw2726

#9 #6 OR #7 OR #8 14047

#10 MeSH descriptor: [Anastomotic Leak] explode all trees 332

#11 MeSH descriptor: [Digestive System Fistula] explode all trees 776

#12 (anastomotic leak*):ti,ab,kw OR (anastomotic fistula*):ti,ab,kw OR (postoperative leak*):ti,ab,kw OR (surgical fistula*):ti,ab,kw OR (digestive fistula*):ti,ab,kw 7808

#13 (dehiscence):ti,ab,kw 3232

#14 #10 OR #11 OR #12 OR #13 10995

#15 (prediction model*):ti,ab,kw OR (risk prediction):ti,ab,kw OR (prognostic model*):ti,ab,kw OR (nomogram*):ti,ab,kw OR (scoring system*):ti,ab,kw 41210

#16 (risk score*):ti,ab,kw OR (validation stud*):ti,ab,kw OR (external validation):ti,ab,kw OR (model performance):ti,ab,kw OR (discrimination):ti,ab,kw 94765

#17 (calibration):ti,ab,kw OR (ROC curve):ti,ab,kw OR (C-statistic):ti,ab,kw 8608

#18 #15 OR #16 OR #17 129077

#19 #5 AND #9 AND #14 AND #18 46

Intuitive table of model predictive factors

Model name	Developer	No. of predictors	Predictors
Logistic regression model	Zhang HF, et al.	6	SIRI ≥1.18, PNI ≥37, tumor located in the middle/lower third of the stomach, operation duration ≥303.5 min, anemia, absence of diabetes
Multivariate logistic regression model	Deng FM, et al.	4	Diabetes, pulmonary insufficiency, preoperative albumin <30 g/L, intraoperative blood loss >400 mL
Nomogram prediction model	Xia ZL, et al.	4	Diabetes, hypoalbuminemia, concomitant pulmonary dysfunction, operation time
Nomogram prediction model	Liu Y, et al.	5	Age ≥60 years, diabetes, BMI >24 kg/m^2, preoperative nutritional risk (NRS2002 ≥3), pulmonary insufficiency
Nomogram prediction model	Man YF, et al.	3	Postoperative day 5: WBC cut-off 11.56 × 10^9/L, CRP cut-off 113.00 mg/L, PCT cut-off 0.841 ng/mL

Model name	Developer	No. of predictors	Predictors
Nomogram and Bayesian network model	Wang YF, et al.	5	Anastomosis method (end-to-side), history of diabetes, preoperative albumin ≤33.6 g/L, history of alcohol consumption, SIRI >1.18
Random Forest (RF) model	Shengli Shao, et al.	10	Hypertension, diabetes, BMI, Brinkman index (smoking), albumin, hemoglobin, tumor size, tumor obstruction, ASA score, operation time
Validation of the existing RF model	Shengli Shao, et al.	10	Hypertension, diabetes, BMI, Brinkman index (smoking), albumin level, hemoglobin level, tumor size, tumor obstruction, ASA classification, operation time
RISK1 (combined clinical–laboratory model) and RISK2 (laboratory-only model)	Xiaodong Tang, et al.	5	Albumin, CRP, PNI, operation time, intraoperative bleeding
Risk-prediction model for anastomotic leakage	Jinrui Wang, et al.	5	Age ≥65 years; preoperative albumin <35 g/L; extent of resection; operation time ≥240 min;

Model name	Developer	No. of predictors	Predictors
			intraoperative blood loss ≥90 mL
LASSO-Logistic model	Wenxiang Ma, et al.	4	CRP within postoperative day 3, age group, history of abdominal surgery, albumin
Nomogram for anastomotic leak	R.-H. Tu, et al.	3	Age ≥65 years; hemoglobin ≤8.0 g/dL; malnutrition
AScore-POD3 based nomogram (Anastomotic Score system on postoperative day 3)	Jinyao Shi, et al.	6	Inflammatory cytokine concentrations in peritoneal drainage fluid: IL-1β, IL-6, IL-10, TNF-α, MMP-2, MMP-9
Support vector machine (SVM) model	Xuanyo Liu, et al.	5	CRP on postoperative day 1/day 4, albumin, NLR, SII, PNI
Risk stratification chart and composite model	Ferdinando Carlo Maria Cananzi, et al.	2	Procalcitonin (PCT) and C-reactive protein (CRP)
Nomogram	Boqi Xu, et al.	5	Smoking history, body mass index (BMI), anastomosis type, blood loss, distance from upper tumor edge

Model name	Developer	No. of predictors	Predictors
Predictive nomogram	Yi Liao, et al.	7	NRS-2002 score, SFMAI (subcutaneous fat/muscle area index), VSR (visceral-to-subcutaneous fat ratio), intraoperative blood loss, operation time, reconstruction type, Lauren classification
LASSO regression risk model	Tiehua Zhao, et al.	2	History of diabetes; Prognostic Nutritional Index (PNI)
